# Challenges in identifying barriers to adoption in a theory-based implementation study: lessons for future implementation studies

**DOI:** 10.1186/1472-6963-12-422

**Published:** 2012-11-23

**Authors:** Andria Hanbury, Katherine Farley, Carl Thompson, Paul Wilson, Duncan Chambers

**Affiliations:** 1 Alcuin C, Department of Health Sciences, University of York, York, England, YO10 5DD, UK; 2 Centre for Reviews and Dissemination, University of York, York, England, UK

**Keywords:** Diagnostic analysis, Tailored implementation, Postnatal depression, Mixed methods

## Abstract

**Background:**

Exploring barriers to the uptake of research based recommendations into practice is an important part of the development of implementation programmes. Techniques to identify barriers can include use of theory-informed questionnaires and qualitative interviews. Conceptualising and measuring theory-informed factors, and engaging health professionals’ to uncover all potential barriers, can be a difficult task. This paper presents a case study of the process of trying to identify, systematically, the key factors influencing health professionals’ referrals for women diagnosed with mild to moderate postnatal depression for psychological treatment. The paper illustrates how the factors were conceptualised and measured and explores the real world challenges experienced, with implications for future implementation studies.

**Methods:**

Theory-informed factors were conceptualised and measured using a questionnaire and interviews. The questionnaire was piloted, before being administered to general practitioners, practice nurses and health visitors working in general practices in one area of the UK NHS. The interviews were conducted with a small sample of general practitioners who had not completed the questionnaire, further exploring factors influencing their referral decisions in the local context.

**Results:**

The response rate to the questionnaire was low (19%), despite selecting the recommendation to target through engagement with local stakeholders and surveying local health professionals, and despite using two reminders, an incentive prize, and phone calls to practice managers to bolster response rates.

**Conclusions:**

Two significant challenges to achieving higher response rates and successfully exploring local context were identified: the difficulties of developing a robust- but feasible- questionnaire to explore theory-informed factors, and targeting recommendations that are important to policy makers, but which health professionals view as unimportant. This case study highlights the “trade-off” between scientifically rigorous collection of data against the pragmatism and flexibility requirements of “real world” implementation. Future implementation studies should explore different ways of identifying factors influencing the adoption of recommendations to bridge this gulf.

## Background

The Translating Research into Practice in Leeds and Bradford (TRiPLaB) research programme is a research and innovation implementation programme funded as part of the NIHR Collaboration for Leadership in Applied Health Research and Care (CLAHRC) for Leeds York and Bradford. TRiPLaB seeks to enhance the health outcomes of the local population through increasing the translation of proven interventions and/or process into practice. TRiPLaB uses a three phase approach to implementation [1]. In the ‘developmental’ phase we select the innovation upon which to focus implementation efforts and explore local contextual factors predicted to influence its adoption. In the ‘implementation’ phase we develop and deliver implementation strategies tailored to the key factors identified. In our ‘evaluation’ phase we employ a mix of qualitative and quantitative research techniques to evaluate the effectiveness, and cost effectiveness, of our tailored implementation strategies.

The developmental phase of TRiPLaB uses theory as a basis for exploring and making sense of the local context for implementation [2]. Due to the variety of different theories that can be applied to implementation, and the consensus that multiple factors at different levels of the health care system [3-7] can influence adoption, TRiPLaB uses a framework derived from a systematic review of dissemination and implementation studies [6]. The framework suggests nine different factors contribute to successful implementation. These include system-level and adopter-related factors, thus, providing broad coverage of potential influences. Six of the factors in the framework can be used to guide detailed exploration of the local context (diagnostic analysis), ensuring all important factors are likely to be covered. These factors are: characteristics of the innovation; system antecedents (structure of the organisation, absorptive capacity for new knowledge, and receptive context for change); system readiness (e.g., tension for change, dedicated time and resources); characteristics of adopters (e.g., motivation, skills); communication and influence (e.g., social networks and peer opinion); and the outer context (e.g., socio-political climate and incentives and mandates). The remaining three factors become more relevant to the subsequent task of developing an implementation strategy, through specifying largely practical factors that should be taken into account: ‘assimilation by the system’ (e.g., this factor emphasises the complexity of achieving adoption at team and system levels, and the often nonlinear nature of this process); the ‘implementation process’ (e.g., human resource issues, external collaboration); and ‘linkage’ (e.g., project management support, user-orientation).

In this paper we focus on the developmental phase of one of TRiPLaB’s case studies. This case study was aimed at increasing general practitioners’, health visitors’ and nurse practitioners’ referrals of women diagnosed with mild to moderate postnatal depression for psychological therapies in one UK NHS Primary Care Trust. The targeted recommendation features in National Institute for Health and Clinical Excellence (NICE) clinical guideline 45 [8]. Psychological treatments available locally for the collaborating Primary Care Trust (PCT) include health visitor listening visits, computerised cognitive behavioural therapy, face-to-face cognitive behavioural therapy, guided self-help, exercise and interpersonal psychotherapy/referral to a counsellor, psychologist or other mental health professional. The paper provides an illustrative case study of designing and using a questionnaire and interviews to measure theoretically and empirically based determinants of innovation adoption in a local context. The main difficulties and challenges encountered in this process are reflected upon and discussed, with the implications outlined for future implementation studies.

## Methods

The research was conducted across 83 general practices within one Primary Care Trust in the North of England, UK. Health professionals invited to participate were those whom the targeted recommendation applied to: general practitioners (N=389), nurse practitioners (N=16) and health visitors (N=52). Ethical approval was granted for this study by National Research Ethics Service (Reference 10/H1311/1).

The underpinning framework for our exploration of the local context [6] contains 69 separate variables, grouped into nine factors; a large number to operationalise and measure. To reduce these to a manageable number, we focussed on those six factors that explore the local context: innovation characteristics; system antecedents; system readiness; characteristics of the adopters; communication and influence and the outer context. Three of the factors – innovation characteristics, outer context and system readiness – however, had already been explored and mapped through three separate activities during the process of selecting the recommendation to target, prior to conducting the diagnostic analysis. The first was a series of meetings with relevant individuals and teams within the PCT to introduce the research project and identify local priority areas and the tension for change in these areas (relevant to the ‘system readiness for change’ factor). The second activity was use of a questionnaire designed to identify those characteristics of recommendations that most strongly influence local health professionals’ prioritisation decisions. This was included to increase the likelihood of selecting a recommendation, based on its characteristics (such as costs and strength of supporting evidence), that local health professionals were more likely to prioritise (relevant to the ‘System readiness for innovation’ and ‘Innovation’ factors in the framework). Finally, pragmatic factors – number of patients the recommendation applied to, availability and robustness of audit data for evaluation purposes, and whether Quality Outcomes Framework targets applied to the recommendation- were checked and mapped to ensure variables specific to the recommendation, and important for safeguarding the robustness of the research, were taken into account (‘System readiness for change’ and ‘Outer context’). Therefore, initial selection of the recommendation was based on a detailed exploration of its characteristics, how health professionals’ themselves prioritise these, degree of prioritisation of the topic in the PCT, and consideration of pragmatic factors.

### The questionnaire

The questionnaire (Additional file 1) was designed to explore the remaining three factors in the framework of relevance to conducting a diagnostic analysis -the adopters, communication and influence, and system antecedents. It contained 42 items across four sections; one for each of the factors plus a demographic section. Given the lack of formal operational definitions and guidance for measuring some of the factors in the underpinning framework, the framework was used as a tool to ‘illuminate the problem and raise areas to consider’ for our diagnostic analysis, as suggested by the frameworks authors (6, p 613). This required us to make a number of decisions regarding how to conceptualise and measure the three factors, outlined below.

#### ‘Adopters’

The adopter-related factors highlighted in the framework are ‘needs’, ‘motivation’, ‘values and goals’, ‘skills’, ‘learning style’ and ‘social networks’. No direction is given to guide researchers wishing to explore ‘adopters’as part of a diagnostic analysis, nor formal definitions of the factors. It was already planned to explore social networks with reference to the communication and influence factor in the framework (see below), highlighting an area of overlap in the framework noted by other researchers [7]. Motivation was conceptualised as motivation to adopt the recommendation, and was operationalised through a single, self-report, measure of health professionals’ intention to refer women for psychological therapies. We took this approach as the psychological literature portrays intention as encompassing motivational factors and as a proximal predictor of actual behaviour in the theory of planned behaviour [9-11], a theory previously used in implementation studies [12,13]. The item was worded: ‘Thinking of women who might present with mild to moderate postnatal depression over the next six months, how likely is it that you would refer them for psychological treatment as a first stage intervention?’ The response was scored on a Likert scale ranging from ‘very unlikely’ [1] through to ‘very likely’ [5]. A set time frame of six months was specified as set time frames are associated with greater accuracy in self-report measures [14].

For the remaining factors relating to ‘adopters’, rather than try and develop measures of ‘needs’, ‘values and goals’, ‘skills’ and learning styles (the latter of which applies more to the design of the behaviour intervention rather than the diagnostic analysis), we decided to measure health professionals’ perceptions of the recommendation across five characteristics. These were: strength of supporting evidence, predicted impact on patient care and clinical outcomes, cost associated with the new way of working, variability in current practice, and whether it is considered to be of national or local importance. These attributes have been suggested as important for successful implementation [7], and matched the characteristics of recommendations measured in the questionnaire completed by health professionals to help select our targeted recommendation. Measuring the same characteristics in both questionnaires enabled us to examine perceptions of characteristics found to be most influential in health professionals’ innovation prioritisation decisions in our earlier survey. An additional attribute – whether there is local expertise in providing psychological therapies for postnatal depression or not- was also included following consultation with NHS colleagues based on their experience of previous quality improvement initiatives that had suggested this to be important. To complete this section of the questionnaire, respondents were required to select which one of two-to-three response options they believed to be the most accurate for each characteristic, with these response options mirroring those used in the earlier questionnaire. For example, for ‘national or local importance’, the response options were ‘local’, ‘national’ and ‘both local and national’.

#### Demographics

Respondents were asked about their job role, number of years since qualifying, number of years in current post, degree of expertise in the area of postnatal depression and the percentage of their patients who are women of child-bearing age.

#### Communication and influence

Communication and influence amongst health professionals was measured through inclusion of social network questions. Social networks and opinion leaders are recognised as an influencing factor upon innovation uptake [6,15,16]. We aimed to capture information networks within the organisation and to identify individuals who were key to information flow on the topic (opinion leaders). Informal diffusion through social network channels is beneficial as interventions using opinion leaders have been shown to successfully promote evidence based practice [17].

We used a sociometric method to gather information on opinion leaders from social networks [16]. Respondents were asked to nominate colleagues they felt to be particularly influential in their day-to-day practice in the clinical management of women with postnatal depression. Of the four dominant methods of collecting opinion leader data - sociometric, key informant, self-designating and observation [15] - sociometric methods have been shown to provide high quality data [18] and their use has been explored in similar studies [16]. Alternative methods such as self-designating techniques do not necessarily identify individuals who are perceived as opinion leaders by their colleagues. It is important to identify individuals who have characteristics in common with colleagues - homophily – as this enables more communication [19]. To understand the characteristics of members of networks and of potential opinion leaders, respondents were also asked to specify whether those named were part of their team and whether they were typically sources or recipients of information.

#### ‘System antecedents’

System level factors were to be measured through inclusion of the validated competing values framework [20] which measures staff climate, leadership style, what bonds staff together, and prioritization of goals. However, the team climate inventory (TCI) [21] was selected over the competing values framework due to its focus on teams rather than the overall organisation; this was considered important because within large organisations, such as the NHS, the combination of different roles, subcultures and hierarchical levels can reduce consensus on what constitutes organisational culture [21]. Furthermore, the CVF has been suggested at be potentially too broad an instrument to inform intervention design [22]. The original TCI tool comprises 38 items grouped into four scales - vision, participative safety, task orientation and support- derived from the four factor theory of work group innovation [21,23] that are proposed to predict team climate for innovativeness. However, we used the shorter, validated, 14 item version of the instrument [24] to reduce respondent burden.

#### Questionnaire administration

The questionnaire was piloted with one GP practice and one Specialist Nursing Team (total N=29) from another PCT, with health professionals requested to advise on ease of completion and the wording of items. Feedback was received from 12 health professionals. Revisions were made to question wording based on feedback before the final version was administered to general practitioners, nurse practitioners and health visitors based in GP surgeries in the collaborating PCT. A protocol for optimising recruitment was developed and used based on Cochrane guidance [25,26]: the first administration was electronic, with the questionnaire sent as a link inside a personalised email sent by the Medical Director. This was followed by an electronic, and then a paper-based reminder to non-responders one and two weeks later respectively, signed by the Deputy Director of Clinical Quality who was responsible for quality improvement work in the PCT. The paper based reminder was accompanied by a paper copy of the questionnaire and a stamped return address envelope for ease of return. Phone calls were made by the quality improvement team in the PCT to practice managers three weeks after the initial mailing to further encourage completion. An incentive prize draw for store vouchers to the value of one hundred pounds was offered, and a promise to provide anonymised feedback on the questionnaire results.

### Qualitative interviews

The qualitative interviews were conducted with a small sample of health professionals to explore their perception of barriers to making referrals, their awareness, knowledge and understanding of the NICE guidance on postnatal depression, and suggestions for how referrals could be increased. This enabled any factors that may have been overlooked during the process of selecting the innovation and exploring the local context to be captured. In exploring perceptions of barriers, the interviews provided an opportunity for issues around skills, values, and needs, specified in the ‘adopters’ category of the underpinning framework, to also emerge. General Practitioners were selected from the list of non-responders to the questionnaire, and invited to participate in a short (20 min) semi structured face-to-face interview, through a personalised letter sent to their practice, which was followed-up by a phone call from the local quality improvement team. Practice managers were notified before the letters were sent, and a twenty pound store voucher incentive prize was offered to those who participated as a thank you for their time.

The interviews enabled those who had not responded to the questionnaire the opportunity to have their views and perceptions taken into consideration. Seven interviews were conducted. Interviews were audio recorded and transcribed verbatim for analysis. Analysis of the interviews was inductive and used thematic analysis [27]; developing key themes from an initial coding, followed by indexing of transcripts. One researcher developed initial key themes and sub-categories from a review of two transcripts. A third transcript was then indexed by three researchers for inter-rater reliability checking. Coding themes were amended and updated to clarify any further themes. Each researcher was then allocated two or three transcripts to code and produced a short paragraph describing the findings in the interview. One member of the team then reviewed and combined the key findings.

## Results and discussion

The response rate to the questionnaire was low (19%, N=86). The first administration of the questionnaire achieved an initial response rate of 7% (N=32). The first, electronic, reminder was followed by an increase of 18 responses (21% of the respondents who received the reminder responded), and the second, paper based, reminder (sent with a paper copy of the questionnaire) was followed by an increase of 36 responses (24% of the respondents who received the second reminder responded). Completion rates were also variable across questionnaire sections; the ‘system-antecedent’ section was highest (19%, N=85), followed by the ‘adopters’ section (15%, N=69) and the ‘communication and influence’ section (13%, N=58). Due to the low response rate the data was unsuitable for a planned two stage analysis [28]. Instead, a series of exploratory, bivariate, analyses were run, focussing on effect sizes to aid interpretation of the meaningfulness of the findings [29] due to the reduced power of the analyses. The qualitative and quantitative findings were then synthesised to arrive at a final set of factors considered to be influential upon health professionals’ referrals for women diagnosed with mild to moderate postnatal depression. The analyses, a summary of the findings, and an overview of the data synthesis are available in Additional file 2 for reference. However, the focus of the rest of the paper is upon the challenges and complexities encountered in the process of conducting the diagnostic analysis, a consideration of the key issues arising from this, and recommendations for future implementation studies.

The low response rate clearly illustrates the challenge of eliciting the views of health professionals to inform implementation research; particularly where research uses questionnaires to try and systematically and rigorously explore factors influencing innovation adoption. Any initial lack of engagement by the health professionals in the developmental stage of an implementation study has significant implications, reducing the robustness and representativeness of the diagnostic analysis. This is critical as the subsequent behaviour-change intervention is then developed to target the barriers identified from the diagnostic analysis. Low response rates when exploring the local context, therefore, make an implementation study vulnerable to overlooking some important barriers and of only capturing the perceptions of those health professionals’ who are especially interested in the clinical topic. Barriers identified from such health professionals may be different to barriers experienced by those who do not respond to the questionnaire; for example, the latter may hold more negative perceptions of the recommendation. This has potential implications for the implementation strategy, which may only target those barriers which apply to more motivated health professionals.

We attempted to maximise responses to the questionnaire by following recommendations in the literature, including the use of two reminders, the second of which was accompanied by a paper copy of the questionnaire, conducting follow-on phone calls, and offering an incentive prize draw [25,26]. The second reminder, accompanied by a paper copy of the questionnaire, had a greater impact than the first, electronic, reminder, suggesting that some of the health professionals preferred a paper-based over an electronic questionnaire. Given the strategies used to bolster response rates, two main explanations are suggested as to why the response rate was still low. First, the topic of postnatal depression may not have had salience with the majority of the health professionals, with topic salience a key variable influencing response rates [26]. At the start of the research, two separate activities were undertaken to try and ensure that we selected a recommendation to target that was prioritised locally by stakeholders and health professionals, to encourage local buy-in and engagement with the diagnostic analysis. First, a series of meetings were held with stakeholders to identify local priority areas. In addition, a questionnaire was run with local health professionals to explore which characteristics of hypothetical recommendations are most influential upon their prioritisation decisions, to guide us in selecting a recommendation that met those criteria. Combined, it was envisaged that these two processes would ensure that we selected a recommendation that, whilst not well adopted at baseline, would have a greater chance of success through PCT and adopter level support. The finding from that questionnaire was that health professionals’ prioritise in particular those recommendations that have a higher impact on patient care and a stronger supportive evidence base, but attach less importance to the costs associated with the recommendation. However, when it came to selecting the recommendation from a shortlist developed from the meetings conducted with local stakeholders, none of the recommendations identified as a local priority exactly met these challenging criteria. Referrals for psychological treatment for women diagnosed with mild to moderate postnatal depression were rated by the team, having reviewed NICE documentation [8], as having a ‘moderate’ evidence base and a ‘moderate’ impact on patient care, rather than a ‘significant’ impact and ‘strong’ evidence base.

This highlights the challenge of marrying what the health professionals- the adopters- consider to be priorities with what stakeholders, such as commissioners, regard as priorities. Trying to engage health professionals at the start of an implementation topic who may not be overly motivated by ‘moderate’ impact and ‘moderate’ evidence base is likely to be a hurdle for other implementation studies. Even if a recommendation does meet such criteria, if health professionals do not perceive it to do so, the challenge still remains: whilst the behaviour change intervention can target negative perceptions of a recommendation to try and encourage uptake, gaining health professionals’ engagement in the initial diagnostic analysis in such instances is still a challenge. However, studies need to continue to target innovations and recommendations that have low adoption levels at the outset, to make best use of resources and avoid potential “ceiling” effects in adoption.

The second explanation for the low response rate relates to the design of the questionnaire; particularly its length and ordering of sections. The questionnaire was long, comprising four separate sections spread over 14 pages in the paper version. Research regarding the effect of longer versus shorter questionnaires on response rates is equivocal, but suggests that questionnaires on highly salient topics can be longer than those on less salient topics [26]. Given our suggestion that the topic may not have been salient to the health professionals, questionnaire length was likely to be particularly influential upon response rates. To try and keep the questionnaire as short as was possible, we opted to use the shortened version of the team climate inventory rather than the long version, and we presented this before the other two sections, which required more detailed and considered responses that we anticipated could deter potential responders if presented first. It is possible, however, that having the more abstract set of questions at the start of the questionnaire, exploring perceptions of team climate, may have negatively impacted upon the response rate, lacking face validity and, again, salience to the health professionals.

The finding that responses were lowest to the communication and influence section – the second rather than the final section- suggests that the content of that particular section also likely impacted upon the response rate. Requesting the health professionals to name colleagues and specify whether they give them advice, seek their advice, and the modalities used to do so may have appeared unusual to the health professionals, and they may have been uncomfortable naming individuals they work with. Unfortunately this is an essential prerequisite for using social network analysis to identify channels of communication and local opinion leaders. In a study exploring the feasibility of involving opinion leaders in implementation efforts [16], health professionals who completed a sociometric measure to identify opinion leaders- as used in this study – reported in interviews afterwards that they had struggled with the concept of opinion leaders and found the questionnaire rather abstract. In the same study, it was also found that opinion leaders varied across clinical topics- and that only 32% of respondents cited the identified opinion leaders. This suggests that the same population would need to be re-surveyed for every new implementation topic to identify opinion leaders, and that the persuasive powers of opinion leaders could be restricted to only a small proportion of the target population when it comes to developing the behaviour-change intervention. With recommendations for future research to harness the potential of social networks to bring about change in practice [30], attention needs to be given to exploring different techniques that can be used to map social networks in the healthcare setting to overcome the response rate challenge and reluctance to name colleagues.

Piloting of the questionnaire with a small sample of health professionals produced few comments regarding the communication and influence section as a whole, nor regarding the decision to place the system antecedents section at the start. This highlights the importance of conducting more detailed assessments of how targeted health professionals feel about the content (rather than just the wording) of implementation questionnaires and exploring their likelihood of responding to such measures. Techniques such as cognitive interviewing [31] can help illuminate issues with questionnaires and encourage a deeper, more systematic, piloting than sending a questionnaire out to a sample and obtaining general feedback regarding wording of items and layout.

The original plan for data analysis had been to run a multilevel model on the questionnaire data [28], with separate analysis of the interview data, delineating the effects of characteristics of the health professionals from team characteristics, thus gaining an understanding of the influence these two levels had upon health professionals’ intention, and knowledge of whether it may be more effective to target teams or individual attitudes with the implementation strategy. The focus of our analyses instead was upon exploring patterns and associations between factors and health professionals’ intentions to refer. Missing the opportunity to simultaneously explore the relative importance of team versus adopter level variables using multilevel modelling was disappointing. One study [32] obtained a significantly higher response rate for a lengthy implementation questionnaire exploring factors at the adopter, team and organisational level. However, in that study ninety-nine general practices registered on a Medical Research Council General Practice Research Framework were sampled: as noted by the authors, these practices are research orientated and can receive funding to support their participation in research studies. The study authors reported involving these practices due to their previous experience of low response rates to implementation questionnaires. The MRC GP research practices database is now discontinued; however, adopting this sort of approach to recruitment is arguably more suited to large scale studies across multiple sites, rather than collaborative implementation studies between single organisations and academic institutions. Given that implementation research occurs in busy healthcare settings, such as hospital wards or GP practices, and needs to develop an understanding of local contextual factors influencing innovation adoption, recruiting enough health professionals in such units to achieve a large enough sample size for more complex statistical techniques such as multilevel modelling will always be challenging, regardless of topic salience, or questionnaire content or length. Adopting a case study approach instead, pulling together multiple strands of evidence from different sources (in our example, a questionnaire and set of interviews), may represent a more realistic, pragmatic and feasible approach.

This case study experienced a low response rate to the questionnaire, despite early stage engagement with local health professionals and stakeholders; piloting the questionnaire; and using evidence-based strategies to bolster response rates. As such, the robustness, representativeness and generalisability of the findings from the questionnaire, and subsequent recommendations for the behaviour change intervention, are limited. The underpinning framework for our diagnostic analysis also presented a challenge; with so many factors proposed to influence innovation adoption, a lack of formal definitions provided, and no specification of cause and effect amongst the factors, a number of decisions had to be made regarding which factors to operationalise (due to the burden of response for busy health professionals if all were operationalised) and how to operationalise them. Another team of researchers [33] recently undertook a systematic literature review to identify existing measures of the same factors from the framework, and where none existed, to devise their own. This was a different approach to ours, which used the framework as an ‘aide memoir’ rather than attempting to measure every factor. Different interpretations of the framework across these two studies are apparent, including our conceptualisation of ‘motivation’ in the ‘adopters’ category as ‘intention to adopt the recommendation’ compared with their ‘readiness to change’: both interpretations coming from the psychological literature, both justifiable. Whilst the work of Cook et al. is valuable as a first step towards operationalising the framework and trying to develop it into a testable theory, the number of factors and competing ways of operationalising them will likely always be a challenge for those wishing to use it.

Future research should explore other techniques that can be used to conduct a diagnostic analysis, to safeguard implementation studies from low initial engagement from health professionals. The consensus that a diagnostic analysis of the local context is important; that different factors will be important across different recommendations and settings; and the high number of recommendations targeted at health professionals, makes the feasibility and sustainability of survey-based approaches to implementation in health services questionable, particularly for topics of low saliency to health professionals. The need to ensure that behaviour-change is a scientific endeavour, adopting a rigorous approach, may struggle to be realised as repeated surveying of health professionals makes their continued use less feasible and increasingly unsustainable. Whilst this approach may be successful for individual research studies, as a technique for future roll-out across the health services to increase implementation of recommendations on an on-going basis, it may be less realistic. Future research should seek to explore and experiment with different methods for exploring the local context, marrying the need for rigour with approaches that are feasible in the long as well as short term.

## Conclusions

Often the ‘diagnostic’ phase of an implementation strategy is hidden, implicit, the rigour hard to discern. Our approach was to explicitly operationalise theoretical constructs, rigorously and systematically gathering and synthesising data from a variety of sources in a transparent way. Response rates were low despite our best efforts, possibly reflecting the complexity of the questionnaire; the competing pressures on health professionals’ time; and a mismatch between the priorities of the PCT and those of health professionals. This case study suggests that the science and practice of building components of influential theories of innovation adoption such as social networks, adopter characteristics and ‘culture’ into planned strategy still has some way to go before ‘off the shelf’ solutions are available to non- academic users seeking to foster innovation adoption in health services.

## Abbreviations

PCT: Primary Care Trust; TCI: Team climate inventory; TRiP-LaB: Translating Research into Practice in Leeds and Bradford; NICE: National Institute for Health and Clinical Excellence; CBT: Cognitive behavioural therapy.

## Competing interests

The authors declare that they have no competing interests.

## Authors’ contributions

AH contributed to the design of this study and the questionnaire and interview schedule, contributed to data analysis and drafted the manuscript. KF contributed to the design of this study and the questionnaire and interview schedule, contributed to data analysis and commented on the manuscript. CT contributed to design of this study, advised on the questionnaire design and helped draft the manuscript. PW contributed to design of the study protocol, advised on the questionnaire design, and provided comments on the manuscript. DC contributed to qualitative data analysis and provided comments on the manuscript. All authors read and approved the final manuscript.

## Pre-publication history

The pre-publication history for this paper can be accessed here:

http://www.biomedcentral.com/1472-6963/12/422/prepub

## Supplementary Material

Additional file 1 Paper copy of questionnaire. Click here for file

Additional file 2 Summary of analyses and key findings. Click here for file
