# Supplementary material for: Challenges in identifying barriers to adoption in a theory-based implementation study: lessons for future implementation studies
Source: BMC Health Serv Res. 2012 Nov 23;12:422. doi: 10.1186/1472-6963-12-422 (PMC3537535; doi:10.1186/1472-6963-12-422)
Supplement: Additional file 1 — Paper copy of questionnaire. [file 1472-6963-12-422-S1.pdf]

THE UNIVERSITY of York

NHS
Bradford and Airedale

Innovations in Healthcare

This survey is designed to explore the factors that influence the uptake of innovations into practice as part of a National Institute for Health Research funded programme. The survey is made up of four short sections. In the first we are interested in the way in which your practice works. The second aims to understand the channels of communication across the PCT, and the third is interested in clinical practice in your field. The final set of demographic questions helps us understand the results of our analysis. Please be assured that they will not be used to identify you at any stage, instead they will be analysed at a group level. There are no 'wrong' answers so please be as honest as possible.

Please be assured that your data will be treated with the strictest of confidence. Although section two asks you to identify individuals by name, this information will be used only during analysis and will not be revealed when results are reported.

The survey has been designed to be completed in the order in which the sections are presented and should take around 20-30 minutes to complete. In recognition of this, on completion of the survey we would like to offer you the chance to be entered into our prize draw for £100 worth of Amazon.co.uk vouchers.

PLEASE READ THE ACCOMPANYING INFORMATION SHEET BEFORE COMPLETING THE SURVEY

Having read the information sheet, if you would like to help us by completing this survey, please tick the box below to indicate that you have read and understood the information sheet and that you consent to your data being used for research purposes.

I have read and understood the information sheet

☐

Section One

Where You Work

There are a number of ways in which the team in which we work can influence how we ourselves work. In the first section of the survey we are interested in your perceptions of the culture, or '*the way things are done around here'*, of your practice.

For each question please circle the response that best reflects your personal opinion.

1 How frequently do you interact with other members of your practice?

Daily	A few times a week	A few times a month	Around once a month	Less frequently than once a month

Thinking about what your practice's day-to-day objectives are, please circle the appropriate response to the following questions.

2 How far are you in agreement with your practice's objectives?

Not at all	To a small extent	Neither to a small nor large extent	To a large extent	Completely

3 To what extent do you think your practice's objectives are clearly understood by other members of the practice?

Not at all	To a small extent	Neither to a small nor large extent	To a large extent	Completely

4 To what extent do you think your practice's objectives can actually be achieved?

Not at all	To a small extent	Neither to a small nor large extent	To a large extent	Completely

5 How worthwhile do you think these objectives are to the organisation?

Not at all	To a small extent	Neither to a small nor large extent	To a large extent	Completely

Thinking of the way that your practice works, please respond to the following statements.

6 We have a 'we are in it together attitude'

Strongly disagree	Disagree	Neither agree nor disagree	Agree	Strongly agree

7 People keep each other informed about work related issues in the practice

Strongly disagree	Disagree	Neither agree nor disagree	Agree	Strongly agree

8 People feel understood and accepted by each other

Strongly disagree	Disagree	Neither agree nor disagree	Agree	Strongly agree

9 There are real attempts to share information throughout the practice

Strongly disagree	Disagree	Neither agree nor disagree	Agree	Strongly agree

10 Are practice members prepared to question the basis of what the practice is doing?

To a very small extent	To a small extent	Neither to a small nor large extent	To a large extent	To a very large extent

11 **Does the practice critically appraise potential weaknesses in what it is doing in order to achieve the best possible outcome?**

To a very small extent	To a small extent	Neither to a small nor large extent	To a large extent	To a very large extent

12 **Do members of the practice build on each other's ideas in order to achieve the best possible outcome?**

To a very small extent	To a small extent	Neither to a small nor large extent	To a large extent	To a very large extent

13 **People in this practice are always searching for fresh, new ways of looking at problems**

Strongly disagree	Disagree	Neither agree nor disagree	Agree	Strongly agree

14 **In this practice we take the time needed to develop new ideas**

Strongly disagree	Disagree	Neither agree nor disagree	Agree	Strongly agree

15 **People in the practice cooperate in order to help develop and apply new ideas**

Strongly disagree	Disagree	Neither agree nor disagree	Agree	Strongly agree

16 To what extent do you feel that you *identify* with your practice?

Not at all	To a small extent	Neither to a small nor large extent	To a large extent	Completely

Please turn over to complete the next section

Section Two **COMMUNICATION NETWORKS**

In this section of the survey we are interested in the people you have had contact with over the past 12 months about your day to day practice in the treatment of depression, specifically, women with depression. We ask this information because sharing information with colleagues is seen as a way of spreading good practice and diffusing new ideas. In the following table please list the names of people with whom you have discussed your day to day practice in the treatment of women with depression. These may be people from whom you have sought guidance or information, people who have sought guidance or information from you, or simply people with whom you have generally discussed practice in this area. We have provided room for six people’s names, however, do not feel that you have to fill all of the boxes.

In the table please write the full name of the people with whom you discuss your practice in the treatment of women with depression. For each of the people you have named, please indicate

- **Who** typically provides advice (you do, the other person does, it is equally likely to be either of you)
- **How often** you are in contact about this topic (whether you feel it is regular versus occasional)
- How do you **typically contact** each other (face to face, in a meeting or training forum, on the phone, by email, other)
- Their **job role** or title
- Whether the contact is (part of your immediate team, part of your extended team, works within the PCT but not in your team, is someone who works outside the PCT)

	Full name	During the contact with this person are you	Frequency of contact	Predominant mode of contact	Their relationship to you?	Job role	
**Person 1**		Usually giving them advice<br><br>Usually receiving their advice<br><br>It goes both ways	Regular<br><br>Occasional	Casual/Face2face<br><br>Telephone<br><br>Email<br><br>Training event<br><br>Formal group event<br><br>Other	Part of your immediate team<br><br>Extended team<br><br>Within the PCT but not within your team<br><br>Outside the PCT	GP<br><br>Practice nurse<br><br>Midwife<br><br>Practice Manager<br><br>Nurse Practitioner<br><br>Commissioner<br><br>Health visitor	Support worker<br><br>Healthcare assistant<br><br>Psychologist<br><br>Community Psychiatric Nurse<br><br>Psychological therapist (covering CBT, counselling)<br><br>Psychiatrist<br><br>Other
**Person 2**		Usually giving them advice<br><br>Usually receiving their advice<br><br>It goes both ways	Regular<br><br>Occasional	Casual/Face2face<br><br>Telephone<br><br>Email<br><br>Training event<br><br>Formal group event<br><br>Other	Part of your immediate team<br><br>Extended team<br><br>Within the PCT but not within your team<br><br>Outside the PCT	GP<br><br>Practice nurse<br><br>Midwife<br><br>Practice Manager<br><br>Nurse Practitioner<br><br>Commissioner<br><br>Health visitor	Support worker<br><br>Healthcare assistant<br><br>Psychologist<br><br>Community Psychiatric Nurse<br><br>Psychological therapist (covering CBT, counselling)<br><br>Psychiatrist<br><br>Other

	Full name	During the contact with this person are you	Frequency of contact	Predominant mode of contact	Their relationship to you?	Job role	
**Person 3**		Usually giving them advice<br><br>Usually receiving their advice<br><br>It goes both ways	Regular<br><br>Occasional	Casual/Face2face<br><br>Telephone<br><br>Email<br><br>Training event<br><br>Formal group event<br><br>Other	Part of your immediate team<br><br>Extended team<br><br>Within the PCT but not within your team<br><br>Outside the PCT	GP<br><br>Practice nurse<br><br>Midwife<br><br>Practice Manager<br><br>Nurse Practitioner<br><br>Commissioner<br><br>Health visitor	Support worker<br><br>Healthcare assistant<br><br>Psychologist<br><br>Community Psychiatric Nurse<br><br>Psychological therapist (covering CBT, counselling)<br><br>Psychiatrist<br><br>Other
**Person 4**		Usually giving them advice<br><br>Usually receiving their advice<br><br>It goes both ways	Regular<br><br>Occasional	Casual/Face2face<br><br>Telephone<br><br>Email<br><br>Training event<br><br>Formal group event<br><br>Other	Part of your immediate team<br><br>Extended team<br><br>Within the PCT but not within your team<br><br>Outside the PCT	GP<br><br>Practice nurse<br><br>Midwife<br><br>Practice Manager<br><br>Nurse Practitioner<br><br>Commissioner<br><br>Health visitor	Support worker<br><br>Healthcare assistant<br><br>Psychologist<br><br>Community Psychiatric Nurse<br><br>Psychological therapist (covering CBT, counselling)<br><br>Psychiatrist<br><br>Other

	Full name	During the contact with this person are you	Frequency of contact	Predominant mode of contact	Their relationship to you?	Job role	
**Person 5**		Usually giving them advice<br><br>Usually receiving their advice<br><br>It goes both ways	Regular<br><br>Occasional	Casual/Face2face<br><br>Telephone<br><br>Email<br><br>Training event<br><br>Formal group event<br><br>Other	Part of your immediate team<br><br>Extended team<br><br>Within the PCT but not within your team<br><br>Outside the PCT	GP<br><br>Practice nurse<br><br>Midwife<br><br>Practice Manager<br><br>Nurse Practitioner<br><br>Commissioner<br><br>Health visitor	Support worker<br><br>Healthcare assistant<br><br>Psychologist<br><br>Community Psychiatric Nurse<br><br>Psychological therapist (covering CBT, counselling)<br><br>Psychiatrist<br><br>Other
**Person 6**		Usually giving them advice<br><br>Usually receiving their advice<br><br>It goes both ways	Regular<br><br>Occasional	Casual/Face2face<br><br>Telephone<br><br>Email<br><br>Training event<br><br>Formal group event<br><br>Other	Part of your immediate team<br><br>Extended team<br><br>Within the PCT but not within your team<br><br>Outside the PCT	GP<br><br>Practice nurse<br><br>Midwife<br><br>Practice Manager<br><br>Nurse Practitioner<br><br>Commissioner<br><br>Health visitor	Support worker<br><br>Healthcare assistant<br><br>Psychologist<br><br>Community Psychiatric Nurse<br><br>Psychological therapist (covering CBT, counselling)<br><br>Psychiatrist<br><br>Other

Section Three Management of Women with Postnatal Depression

In the previous section of the survey you were asked about who you contact, or who contacts you for guidance and information relating to the treatment of women with depression. In this section of the survey we are interested in your views on referrals for psychological treatments as a first stage intervention specifically for mild to moderate postnatal depression (covering any depression diagnosed within 12 months following birth).

When we refer to 'psychological treatments' we mean *any* non-pharmaceutical treatment for postnatal depression: Cognitive Behavioural Therapy (CBT), Computerised CBT, other guided self help, exercise, Interpersonal Psychotherapy, non-directive counselling delivered at home (Listening visits), and referral to a counsellor, psychologist or other mental health practitioner.

23 Are you aware of the NICE guidance on the use of psychological treatments for mild to moderate postnatal depression?

Yes   No

24 If 'yes', how familiar would you say you are with the guidance?

I am aware of it but have not read it   I have read it   I have read and used it in clinical practice

25 Thinking of women who might present with mild to moderate postnatal depression over the next six months, how likely is it that you would refer them for psychological treatment as a first stage intervention?

Very unlikely   Unlikely   Neither unlikely nor likely   Likely   Very likely

Thinking of referrals for psychological treatments as a first stage intervention for mild to moderate postnatal depression, for each of the questions below, please circle the response that best reflects your personal opinion:

26 In your opinion, would you rate the impact on patient care and clinical outcomes of such referrals as being of

Limited improvement	Modest improvement	Significant improvement

27 In your opinion, would you rate the costs associated with such referrals as being

Small	Medium	Large

28 In your opinion, do you feel that there is local expertise in the field relating to such referrals?

Yes	No

29 In your opinion, do you feel that current services are meeting minimum standards in terms of such referrals?

Yes	No

30 In your opinion, the strength of the supporting evidence for such referrals is

Weak	Medium	Strong

31 **In your opinion, do you consider such referrals to be a**

National priority | Local priority | Both national and local priority

32 **In your opinion, would you rate the prevalence of postnatal depression in the local population as being**

low prevalence of condition/disease | high prevalence of condition/disease

33 **Thinking back over the past six months, how many women have you seen with mild to moderate postnatal depression? (covering any depression diagnosed within 12 months following birth)**

34 **Thinking back over the past six months, of the women that you have seen with mild to moderate postnatal depression how many have you referred for psychological treatment as a first stage intervention?**

35 **If there have been situations where you have prescribed anti-depressants first, what contributed to your decision to do so?**

36 **Thinking specifically about referring women with mild to moderate postnatal depression for psychological treatments, are there any factors, not covered by the survey so far, that you feel make it more or less easy for you to refer in your day to day practice?**

Section Four **About You**

Please tick the appropriate box for each question

37 Your job role is (Please tick all that apply)

- GP: Partner ☐
- GP: Salaried ☐
- GP: Locum ☐
- GP: Retainer ☐
- GP with Specialist Interest ☐
- GP: PCT Clinical Lead ☐
- Nurse Practitioner ☐

37a **If you indicated that you are a GP with Special Interest or PCT Clinical Lead, please indicate your specialist interest/lead area in the box below**

38 **You work**

Full time Part time

39 **Your number of years since qualifying has been**

40 **The number of years in your current post has been**

41 **How much expertise do you feel that you have in the area of postnatal depression?**

I have no expertise	I have some expertise	I have a lot of expertise

42 **In an average week, approximately what percentage of the patients that you see would you say are females of child bearing age?**

Thank you for taking the time to complete this survey.

We would like to enter you into our prize draw for £100 worth of Amazon.co.uk vouchers as a thank you for taking the time to complete our survey. If you would not like to be included in the prize draw, please tick the box below.

Please do not enter me into the prize draw ☐

If you would like to know more about The TRiP-LaB Project please visit our website at

www.trip-lab.com

Blank Page
