# Supplementary material for: Challenges in identifying barriers to adoption in a theory-based implementation study: lessons for future implementation studies
Source: BMC Health Serv Res. 2012 Nov 23;12:422. doi: 10.1186/1472-6963-12-422 (PMC3537535; doi:10.1186/1472-6963-12-422)
Supplement: Additional file 2 — Summary of analyses and key findings. [file 1472-6963-12-422-S2.pdf]

Additional file 2.pdf

This additional file summarises the key analyses and findings from the questionnaire and interviews. Due to the number of analyses, a tabulated breakdown of the descriptive statistics for each construct, key analyses, significant findings, and their implications for the planned implementation strategy is summarised below.

Analysis of the system antecedents section of the questionnaire

Analysis comprised calculation of mean scale scores for the four scales. We then examined the correlation between scale scores and self-reported referral intention/motivation to refer women with mild to moderate postnatal depression for psychological therapies, to explore the strength of relationship between team climate and self-reported intention to adopt the recommendation. The self-reported intention/motivation measure was used as the main outcome measure in this and analysis of other sections due to previous research which has found self-reported intention to exert a medium to large effect on clinicians' actual behaviour (34), and the use of self-reported intention as an outcome measure in other implementation studies (e.g., 13).

Table 1: summary of analyses on the 'system-antecedents' section of the questionnaire

**Purpose of analysis**	**Description of analysis**	**Results**
Test strength of relationship between team climate scale scores and self-reported intention to refer	Pearson's r correlations between team climate scale scores and self-reported intention	1. No significant correlations were found between TCI scale scores and intention to refer (Vision scale, r=- 0.15, p>0.05; Participative safety r = - 0.13, p>0.05; Task orientation r = - 0.18, p>0.05; Support for innovation r = 0.003, p>0.05).

*r effect size, small = 0.1, medium = 0.3, large = 0.5

As can be seen from table 1, no significant correlations were found between perceptions of team climate across the four TCI scales and intention to refer. The small effect sizes seen for correlations between team vision, participative safety, task orientation, innovation and intention suggest only a weak relationship between perceptions of team climate towards innovation and adoption of the targeted recommendation. No further tests of predictive relationships between the TCI and self-reported intention were run due to the low strength of relationship found in the correlations. This section of the analysis therefore yielded no recommendations for our implementation strategy.

Analysis of the communication and influence section of the questionnaire

For analysis of this section, three new variables were computed from responses:

- Number of contacts reported
- Number of contacts where respondent reported giving advice
- Number of contacts where respondent reported receiving advice.

A list of named contacts was also compiled to try and identify local opinion leaders. Contingency tables and chi square tests of association were used to examine associations between number of contacts reported and individual-level demographic variables, to explore whether having a greater number of contacts is associated with key variables such as degree of expertise in managing postnatal depression.

Table 2: summary of analyses on social networks section of the questionnaire

**Purpose of analysis**	**Description of analysis**	**Results**
Explore for associations between number of social contacts reported by health professionals and individual-level demographic variables	Chi square analyses	2. Having more contacts amongst colleagues (4 or more compared with 3 or less) associated with having greater familiarity with PND guidance $\chi 2$=35.494, df 4, p≤0.0001
Explore relationship between number of social contacts reported by health professionals' and self-reported intention to refer	Chi Square	3.Having more contacts amongst colleagues (4 or more compared with 3 or less) associated with having higher intention to refer $\chi 2$=3.888, df 1, p≤0.5

Over half of the respondents (57%) reported having 3-4 social contacts within their network, with contacts typically within their immediate team (58%) and made casually (71%), suggesting ease and convenience in day to day work largely influence contacts made. The findings in table 2 suggest health professionals who report having greater familiarity with the guidance tend to have more colleagues in their networks. Identification of potential opinion leaders from the social network data was impossible due to low numbers of responses on these items; only six health professionals were cited more than once as a contact by the responders. The implementation strategy may need to promote networking between health professionals to encourage sharing of good practice and learning from peers.

Analysis of adopters section of the questionnaire

Analysis comprised examining the proportion of responses falling into each response option for each of the six characteristics. Respondents' perceptions were then compared with objective ratings across the same characteristics based on discussion between members of

the research team following review of the evidence provided by NICE, combined with local knowledge provided by NHS colleagues (table 3). This approach meant respondent's perceptions could be compared against objective ratings to identify any misperceptions of the recommendation that could influence intention to adopt. Descriptive statistics for the intention/motivation to refer item were computed (table 4). Demographic differences in intention/motivation were then tested using a series of one way ANOVAs, with intention/motivation to adopt the recommendation as the dependent variable (table 5).

Table 3 summary of respondent's and the teams' ratings of psychological treatments across the six characteristics of recommendations proposed to be important for innovation adoption (7)

	**Impact on patient care**	**Cost**	**Strength of supporting evidence**	**Currently meeting minimum standards**	**Priority**	**Expertise**
Summary of research team rating of the 6 different psychological treatments available locally	2 locally available treatments rated as 'limited' (33%)<br><br>4 rated as 'moderate' (67%)	3 treatments rated as 'low' (50%)<br><br>1 treatment rated as 'medium' (17%)<br><br>2 treatments rated as 'high' (33%)	2 treatments rated as 'limited' (33%)<br><br>4 treatments rated as 'moderate' (67%)	All 6 treatments rated 'no: not meeting minimum standards' (100%)	5 treatments rated as 'local and national priority' (75%)<br><br>1 treatment rated as 'local priority' (25%)	All 6 treatments rated as 'yes: local expertise is available' (100%)
Summary of responders' perceptions of psychological treatments overall	'Limited' = 13% of responses<br><br>'Moderate' = 58% of responses<br><br>'Significant' = 29% of responses	Small = 17.6% of responses<br><br>Medium = 64.7% of responses<br><br>Large = 17.6% of responses	Limited = 6.1%<br><br>Moderate = 59.1% of responses<br><br>Strong = 34.8% of responses	Yes = 44.9% of responses<br><br>No = 55.1% of responses	National = 16.9% of responses<br><br>Local and national = 67.7% of responses<br><br>Local = 15.4% of responses	Yes = 72.1% of responses<br><br>No = 27.9% of responses

Table 4: summary of descriptive statistics for intention/motivation measure

**Descriptive statistics**
Mean (3.54)
Standard deviation (1.18)
Skewness (-0.67)
Kurtosis (-0.44)
% of responses ‘very unlikely’ to ‘unlikely’ (21.7)
% of responses ‘neither unlikely nor likely’ (15.9)
% of responses ‘very likely’ to ‘likely’ (62.3)

The average self-reported intention for referring women with mild to moderate postnatal depression for psychological therapy was 3.54 (N=69), with 21.7% of respondents indicating their intention to be either ‘very unlikely’ or ‘unlikely’, and 62.3% indicating it to be ‘likely’ or ‘very likely’. This suggests a moderate degree of intention/motivation to refer women with mild to moderate postnatal depression for psychological treatment.

Table 5: summary of analyses on “adopters” section of the questionnaire

**Purpose of analysis**	**Description of analysis**	**Key findings**
Test for demographic differences in self-reported intention to refer.	One way ANOVAs with intention as dependent variable, and individual level demographic variables as independent variables	4. Those with higher number of cases of PND in their caseload (5 or more, N=28) had a higher average intention score (mean= 3.71) than those with lower number of cases (0-4, N=35) (mean=3.37): $F(1, 61) = 1.21$, $p=0.275$, $\eta^2=0.14$*<br><br>5. Those with ‘some’ self-reported expertise in PND had higher intention (mean=3.55) than those with ‘no expertise’ (mean=3.14): $F(1, 61) = 3.63$, $p=0.399$, $\eta^2=0.1$*
Explore health professionals’ perceptions of psychological treatments for PND against the 7 innovation characteristics and compare with independent ratings by the research team of each psychological treatment available locally to identify which characteristics are perceived most negatively and identify any misperceptions	Examine modal responses for each innovation characteristic and compare with ratings agreed by the team	6. 28% of respondents felt there was no local expertise for the different psychological treatments available locally, whereas the research team considered local expertise to exist for all psychological treatments locally, based on discussion with NHS colleagues.<br><br>Majority of respondents (65%) perceive costs of psychological treatment to be ‘medium’ with lower proportions perceiving costs to be ‘low’ (18%) or ‘high’ (18%) than were rated by research team based on review of the costings of the different psychological treatments that fall under psychological<br><br>Respondents perceived the predicted impact on patient care of psychological treatments to be more positive than the ratings of the different psychological treatments by the research team: 13% of respondents perceived there to be ‘limited impact’ of psychological treatments in general compared with 50% of the treatments being rated as such by the research team based on evidence summarised in the NICE guidance. Furthermore, 29% of respondents perceived impact to be ‘significant’ compared with 0% of the treatment options by the research team

*η^2, small effect = 0.01, medium effect = 0.09, large effect = 0.25

A summary of the respondents' and research teams' rating of psychological treatments across the dimensions suggested to be important for successful implementation is provided in table 4. Respondents' perceptions of psychological treatments available locally and independent ratings of them by the research team differed across some of the dimensions (particularly the costs of different psychological treatments and extent of local expertise). As can be seen in table 5, analysis of this section of the questionnaire also suggests that the greater the familiarity with the guidance the more likely the intention to refer. These findings combined suggest the implementation strategy should focus on developing familiarity with the guidance, emphasising the relative merits of the range of psychological treatments available locally, and shifting perceptions of the costs, and presence of local expertise in the management of PND – perhaps through networking.

Qualitative interviews

Four themes emerged from the analysis:

Familiarity with postnatal depression guidance

Around half the respondents reported receiving but not being familiar with the NICE guidance on postnatal depression. However, almost all respondents had referred women with PND to psychological therapies; even those who did not report doing so. This suggests that the behaviour-change intervention should include strategies to help raise awareness of and familiarity with the postnatal depression guidance.

Lack of awareness of the range of psychological therapies

Health professionals' described only a limited range of psychological therapies. Cognitive behavioural therapy was routinely highlighted as the only psychological therapy but interviewees would then go on to discuss health visitor support ('listening visits') and other forms of counselling. Three GPs displayed a lack of knowledge of any therapy other than CBT. However, two of these were positive toward psychological therapies generally. The theme highlights the importance of raising awareness of the range of locally available services.

The need to overcome practical barriers to accessing psychological therapies

Barriers were important and related to choice. One respondent highlighted the relative absence of barriers to prescribing drugs as opposed to the large numbers associated with referring women for psychological therapies. GPs perceived the key factor limiting referrals to psychological therapies both for PND and for depression in general, to be waiting times for initial assessment and treatment. All psychological treatments were perceived as having waiting lists, but CBT was often singled out. This perceived barrier meant that the two 'positively orientated' GPs referred to in the previous paragraph considered referral as pointless. Again, a limited understanding of what constitutes 'psychological therapies' in the NICE guidance was evident.

The practicalities of accessing psychological therapies as a patient were cited by half the respondents. Reasons given included the necessity to attend formal sessions regularly, unusual and unfamiliar treatment locations, and accessing childcare whilst under treatment. Although, again, the exemplars referred to were overwhelmingly restricted to CBT alone.

Once more, findings suggest that the behaviour-change intervention should raise awareness of the range of services available, as well as combating negative perceptions of limited access and extended waiting times by promoting the range of services and spreading demand.

The preferences and expectations of women with PND

Women were often cited as preferring drug treatments; sometimes this was due to a lack of understanding of other therapies available. Social stigma associated with psychological treatments was another important factor leading to a preference for drugs. One GP described patients in deprived areas as "resistant" to psychological treatments, and yet also reported patients in other (wealthier) areas as specifically requesting Prozac. This finding suggests that the behaviour-change intervention could include a component to target service-user expectations of treatment.

Synthesis of the findings

Results were combined from the questionnaire and interview summaries; from this synthesis emerged three key local contextual factors influencing health professionals' intention to refer women with mild to moderate postnatal depression for psychological treatments. Table 6 summarises the main questionnaire and interview findings, the three key factors identified from the synthesis, and the theory-informed factors that they relate to.

Key factor one: Need to raise awareness of the PND guidance to combat health professionals' lack of familiarity with it, as well as promoting the range of psychological treatments available locally and the relative merits (cost, effectiveness, and waiting list times) of different treatments. This factor relates to the 'adopters' measure in the questionnaire: analysis found that health professionals with more experience of managing PND and those more familiar with the guidance, were more likely to intend to refer women for psychological treatment. The qualitative interviews further suggested a lack of awareness of the range of psychological treatments available locally, and a narrow focus on CBT alone; which was perceived as having a long waiting list.

Key factor two: need to develop local expertise and perceptions of the presence of local expertise in the management of postnatal depression. This factor relates to 'adopters': analysis found that health professionals perceived an absence of local expertise in the management of PND. This factor also relates to 'communication and influence', with analysis of the social network items in the questionnaire indicating that those health professionals who reported having more social contacts tended to have more familiarity with the guidance, and were more likely to intend to refer for psychological treatment.

Key factor three: need to address women's' expectations of treatment and/or equip GPs with the techniques for dealing with expectations of treatment. This factor relates to the 'adopters' and emerged from analysis of the qualitative interviews, with the health professionals perceiving service-users' demands for drug treatment over psychological therapy to be an important influence on their treatment recommendations.

Table 6: summary of how key findings from questionnaire and interviews map onto key factors and theoretical factors

**Result**	**Source**	**Key factor**	**Theoretical factor**
2 Having more contacts within social network associated with greater familiarity with the PND recommendations<br><br>6 Perception of low levels of local expertise in PND management	Questionnaire	Two	Communication and influence<br><br>Adopter
3 Having more contacts within social network associated with higher intention to adopt<br><br>4 Those with higher number of PND cases had higher average intention to adopt score<br><br>5 Those with some self-reported expertise had higher intention to adopt	Questionnaire	One	Communication and influence<br><br>Adopter<br><br>Adopter
Reported familiarity with PND guidance	Interview	One	Adopter
Lack of awareness of the range of psychological therapies	Interview	One	Adopter
Perceived barriers to accessing psychological therapies	Interview	One	Adopter
The preferences and expectations of women with PND	Interview	Three	Adopter
